# ProTstab2 for Prediction of Protein Thermal Stabilities

**DOI:** 10.3390/ijms231810798

**Published:** 2022-09-16

**Authors:** Yang Yang, Jianjun Zhao, Lianjie Zeng, Mauno Vihinen

**Affiliations:** 1 School of Computer Science and Technology, Soochow University, Suzhou 215006, China; 2 Collaborative Innovation Center of Novel Software Technology and Industrialization, Nanjing 210000, China; 3 Department of Experimental Medical Science, BMC B13, Lund University, SE-22184 Lund, Sweden

**Keywords:** protein cellular stability, stability prediction, protein property, machine learning predictor, artificial intelligence, gradient boosting

## Abstract

The stability of proteins is an essential property that has several biological implications. Knowledge about protein stability is important in many ways, ranging from protein purification and structure determination to stability in cells and biotechnological applications. Experimental determination of thermal stabilities has been tedious and available data have been limited. The introduction of limited proteolysis and mass spectrometry approaches has facilitated more extensive cellular protein stability data production. We collected melting temperature information for 34,913 proteins and developed a machine learning predictor, ProTstab2, by utilizing a gradient boosting algorithm after testing seven algorithms. The method performance was assessed on a blind test data set and showed a Pearson correlation coefficient of 0.753 and root mean square error of 7.005. Comparison to previous methods indicated that ProTstab2 had superior performance. The method is fast, so it was applied to predict and compare the stabilities of all proteins in human, mouse, and zebrafish proteomes for which experimental data were not determined. The tool is freely available.

## 1. Introduction

Stability is an essential property for all proteins and other biological macromolecules. Proteins need to be stable in the temperature and conditions where they are active and functional. In addition, stability is the major property that has been tried to improve with protein engineering, mainly to increase the thermal stability [1,2]. Among the effects of disease-related variations, reduced stability is very common [3]. Measurements of stabilities of purified proteins and those in cells are laborious and available data are limited. Therefore, computational solutions have been developed.

There are two major categories of protein stability predictors: those that predict the stability of the entire protein, and those that forecast the effects of sequence changes on stability. Here, the focus is on entire protein stabilities. Several prediction methods have been developed for this purpose based on different types of descriptors, such as sequence lengths [4,5], sequence [6,7], physicochemical [8] and surface [9] features, the living temperature of organism and salt bridges [10], various statistical and sequence potentials [11,12,13], and numerous protein characteristics [14]. For a more extensive description of the types of features and characteristics used in method training, see [14,15].

The field has for long been hampered by the amount of available experimental data. Stability determination is labor intensive and only limited numbers of validated results have been known. In recent years, methods for large-scale stability determination have been introduced. ProTstab [14] was the first machine learning (ML) method in the field and was based on data for cellular stability measurements combining limited proteolysis and mass spectrometry of proteins in four organisms: *Escherichia coli*, *Homo sapiens*, *Saccharomyces cerevisiae*, and *Thermus thermophilus* [16]. There was information for 3520 proteins. More recently, meltome atlas was released with protein stabilities in 13 organisms representing different branches of the tree of life [17]. There were data for about 48,000 proteins. Thermal profiles of proteins were obtained by heating cells or lysates at different temperatures, after which precipitates were removed with centrifugation. The soluble fraction was digested with trypsin and the peptides were separated by offline liquid chromatography and identified and quantified with online liquid chromatography–tandem mass spectrometry (LC–MS/MS). Melting profiles were obtained by combining results across the investigated temperatures. Melting temperature (*T_m_*) means the temperature that shows 50% precipitation or area under the (melting) curve (AUC) for a protein. Test temperatures ranged up to 30 °C above the optimal growth temperature (OGT) of the species.

We combined the two data sets and developed a new gradient boosting-based machine learning method ProTstab2 for protein stability prediction. The method is freely available as a web service. Its performance is superior to ProTstab [14] and other previously published methods. Since the tool is fast, it can be used for various tasks and even with large numbers of proteins. We predicted the stabilities of proteins in human, mouse, and zebrafish proteomes.

## 2. Results

A novel protein stability predictor was developed based on systematic method optimization. The original ProTstab was trained and tested on stability information for 3520 proteins obtained in a cell-wide analysis of thermal unfolding [16]. There were data for four organisms, *E. coli*, *S. cerevisiae*, *T. thermophilus*, and humans. Subsequently, a much wider study for meltome atlas was published [17] for cellular stability of proteins in 13 species. The method is based on heating of cells and subsequent protein extraction and identification with liquid chromatography–tandem mass spectrometry. The optimal growth temperatures of the species ranged from 15 to 70 ℃. The details for the data set and the origin of species are in Table 1. The largest number of proteins (6456) originates from humans, followed by mice (5800), zebrafish (3362) and *Caenorhabditis elegans* (3327).

**Figure 1:**
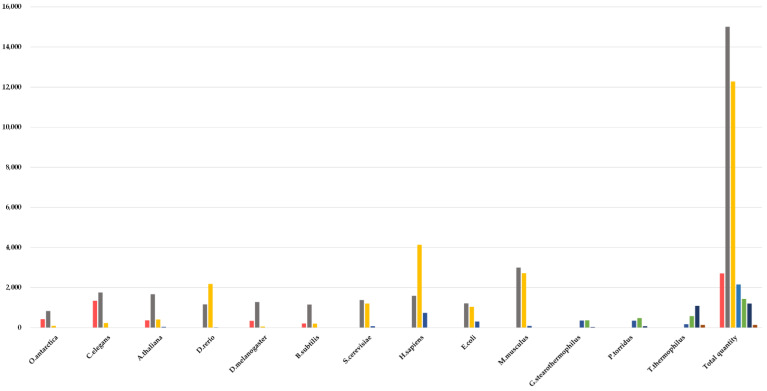
Distribution of *T_m_* values for proteins in each species. Proteins were binned and color-coded as follows: <30 °C pale blue, between 30 and 40 °C red, between 40 and 50 °C gray, between 50 and 60 °C orange, between 60 and 70 °C blue, between 70 and 90 °C green, between 80 and 90 °C dark blue, and over 90 °C brown.

The data from the two sources were combined and duplicates were removed. There were a total of 34,913 proteins. The *T_m_* values ranged from 27.6 °C to 98.9 °C, thus covering the natural distribution of *T_m_* values. The distribution of the melting temperatures for each organism is shown in Figure 1. The organisms were arranged into increasing order based on their optimal growth temperature. The thermal stability of proteins is an intrinsic property [17] and the distribution in organisms follows their growth temperature.

The data set was randomly divided into training, testing, and blind test sets. The distribution of proteins in each species in ProTstab, meltome atlas, and our data set are shown in Table 2.

Previous study [14] indicated that in protein stability prediction there are not such strong features as in variation pathogenicity prediction with PON-P2 [18] or PON-All [19]. To find the most relevant set of features, we collected a wide selection of parameters. The majority of them were calculated with program protr (Supplementary Table S1). In addition, parameters from other programs based on amino acid sequence and sequence context were included. In total, we had 6935 features.

### 2.1. Choice of Algorithm

First, we trained seven regression predictors to choose the best-performing algorithm. The algorithms included decision tree (DT), random forests (RF), support vector regression (SVR), gradient boost regression tree (GBRT), extreme gradient boosting (XGBoost), light gradient boosting machine (LightGBM), and multi-layer perceptron (MLP) regressor. All the features were used for training the predictors, and the performances of the algorithms in 10-fold cross-validation (CV) are presented in Table 3. LightGBM showed the best performance for all the measures: Pearson correlation coefficient (PCC) of 0.75, root mean square deviation (RMSD) 7.11, *R*^2^ 0.56, mean squared error (MSE) 50.50, and mean absolute error (MAE) of 5.27. GBRT, XGBoost, and MLP regressor were the next best algorithms. The performance was the poorest for DT and SVR. LightGBM also had the fastest training time (673 s), about one-tenth of the time of the next fastest algorithm, SVR (5258 s), and only about 3% of the slowest method, RF. LightGBM was chosen to train the final predictor.

### 2.2. Feature Selection and Method Training

We performed extensive feature selection to find the most relevant features and to limit the number of features to a minimum. We applied two feature selection approaches, recursive feature elimination (RFE) and recursive feature elimination with cross-validation (RFECV).

We used RFE to select features, for that purpose, we trained nine regression predictors with the top 50, 100, 200, 300, 500, 1000, 2000, and 3000 features, or with all the 6935 features. The optimal number was 1214 features for the RFECV-based predictor. The performances in the 10-fold CV are shown in Table 4.

In total, five measures were used to chart the full performance of the predictors. Ten-fold CV was used in all the tests. The results for predictors with 200 and 300 features were the best and had almost identical performances, the differences were marginal. We chose the predictor with 200 features as smaller number of features means that the space of possible combinations can be better covered. We call this predictor ProTstab2.

The features used in ProTstab2 are listed in Supplementary Table S2 along with their importance scores in RFE. The scores range from 88 for group 5 amino acid frequency to 5 for the composition of secondary structures of group 1. Most of the importance scores have low scores, but together they contribute to reliable predictions. The selected features originate from a wide range of types. Among the most common features are several quasi-sequence order descriptors, various composition descriptors and scales-based descriptors. There are also several amino acid, dipeptide and amino acid type features.

### 2.3. Performance of ProTstab2 Algorithm

The performance of ProTstab2 was tested on randomly selected blind test data, the results are in Table 5. All the measures are clearly better in comparison to the original ProTstab: PCC is 0.803 vs. 0.736 indicating a substantial improvement. Error measures RMSE, MSE and MAE are improved and show values 9.097 (9.636 for ProTstab), 82.752 (93.581) and 6.934 (8.158), respectively. *R*^2^ measures how the true data fit to the model. ProTstab2 shows a good fit in the blind test data, 0.580 vs. −0.850 for ProTstab.

Of the other methods for protein stability it was possible to compare only the method of Ku and coworkers [7] and SCooP [13]. Ku et al. tool is not a regression predictor, instead, it classifies proteins into three melting point categories (*T_m_* < 55, 55 < *T_m_* < 65, and *T_m_* > 65 °C). To compare to this tool, we submitted our blind test set cases to the web service at http://tm.life.nthu.edu.tw (accessed on 6 September 2022). The classification accuracy of the three categories is 83.8% (2884 proteins correct out of 3443) for ProTstab2 while the Ku et al. predictor had 15.5% correct (532 out of 3443).

SCooP was another program with which ProTstab2 performance could be compared. Unlike ProTstab2, SCooP is structure-based, which restricts its use to only proteins for which structures have been determined. We identified structures for proteins in our blind test data set from Protein Data Bank [20] with Blast [21] and identified 500 proteins that had sequences identities 95% or higher and which covered at least 90% of the length of the protein sequences. These thresholds were used since sequences for PDB entries can be slightly different in comparison to those in sequence databases and since many of the structures are not for the complete proteins. As ProTstab2 has been trained for full-length proteins we set the threshold for sequence coverage to 90%, which should include all the domains in multidomain proteins.

Results in Table 6 show that ProTstab2 is clearly better than SCooP on all the used measures. Error scores are significantly better for ProTstab2. The outcome is interesting as one could think structures to be better starting point for these calculations. Apparently, the used structural features are not discriminative enough for high accuracy.

The results indicate that ProTstab2 has a clear improvement in comparison to ProTstab and SCooP, both of which are superior to the method of Ku et al. The training data for ProTstab2 is about 10 times larger than for ProTstab, it represents many more species, and covers a wide range of *T_m_* values. Combined, these factors contribute to the excellent performance.

### 2.4. Application of ProTstab2 to Proteome-Wide Predictions and Comparison of Stabilities of Human, Mouse, and Zebrafish Proteins

ProTstab2 is a fast method, and it can be used to predict the stability of proteins in any organism. It can be applied also to large-scale studies. We predicted the stability of all human, mice, and zebrafish proteins. In the case of humans, the recommended MANE reference sequences were used [22]. The results can be downloaded from the ProTstab2 website. Proteins with experimental *T_m_* values were excluded. The average predicted *T_m_* values were 50.5 for humans, 50.2 for mice, and 57.9 °C for zebrafish. These species were chosen as they are widely used model systems. Once there is a list of all proteins of interest (even the entire proteome in an organism) the method can quickly calculate the predictions.

**Figure 2:**
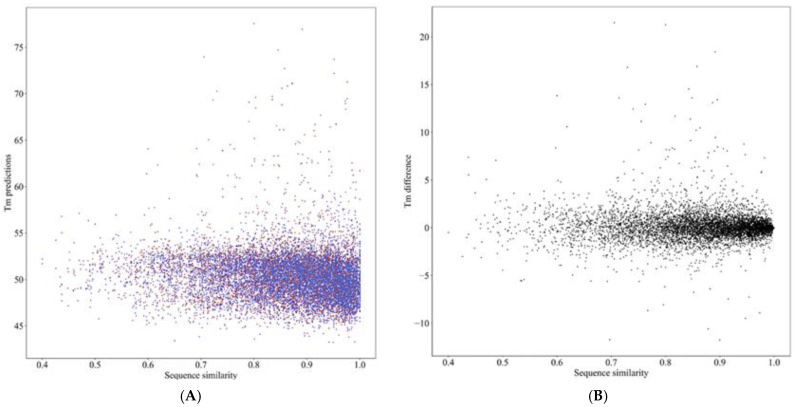
Comparison of predicted stabilities of human and mouse proteins. A total of 6492 orthologous pairs were compared. (**A**) Distribution of predicted *T_m_* values for human (red) and mouse (blue) proteins. (**B**) Difference of the predicted stabilities. Human proteins are on average slightly more stable than mouse proteins.

To highlight the applicability of the method we compared the predicted *T_m_* values for a random selection of 6942 human–mouse ortholog sequence pairs. The orthologs were obtained with biomart from EBI. The distribution of predicted *T_m_* values is shown in Figure 2A. The graph shows the stability along with increasing sequence similarity. Interestingly, the sequence similarity does not correlate at all to the stability (Figure 2B). Although the OGT of both human and mouse is 37 °C, the human proteins tend to be slightly more stable. The results are similar to experimental *T_m_* values in meltome atlas [17].

### 2.5. ProTstab2 Web Application

ProtStab2 is freely available as a web application at http://structure.bmc.lu.se/ProTstab2/ (accessed on 12 September 2022) and at http://8.133.174.28:8000/ProTstab2 (accessed on 12 September 2022). The program uses as input protein sequence(s). ProTstab2 provides complete report which is sent to the user by email when ready. The website contains data sets used for training and testing, as well as the results for the predictions of three proteomes.

## 3. Discussion

Computational tools are needed to predict protein melting temperatures since the data are still largely scarce. Protein stability is of interest as it is integral to numerous properties of proteins in the natural environment and biotechnological processes. Changes to stability are common among disease-related variations [3]. We developed a novel predictor by following a systematic approach [23].

Our previous predictor ProTstab was trained on 3520 proteins from four organism [14]. A new data set with about 48,000 protein *T_m_* measurements [17] was combined with the original data, to sum up to 34,913 unique records. The OGTs of the organisms range from 15 to 70 °C and the *T_m_* values cover a range of 70 degrees. Thus, the training data are representative and can facilitate the development of a reliable predictor. As stability of proteins is an intrinsic property [17], we collected a very large number of protein descriptors. In total, there were 6395 features, see Supplementary Table S1.

We first tested seven artificial intelligence algorithms including DT, RF, SVR, GBRT, XGBoost, LightGBM, and MLP regressor to find which one of them has the best performance when all the features were used for training. To assess the performance of the algorithms, we used five distinct measures. It is essential to use relevant measures and to cover different aspects of performance, see [24,25]. LightGBM was the best algorithm based on all five measures (Table 3). In addition, it was clearly the fastest algorithm to train.

Next, we trained a predictor with LightGBM and tested two feature selection approaches, RFE and RFECV (Table 4). RFECV optimizes the number of features, in this case, it was 1217 features. For RFE the stop criterion has to be given, we tested 9 selections ranging from 50 to the full set of 6935 features. The results for 200 and 300 features showed the best performance and better scores than a method trained based on RFECV-selected features. The most important features represent various types of protein descriptors (Supplementary Table S2).

The LightGBM algorithm is powerful, but it can be overfitted. To avoid this, we chose for the final method 200 RFE selected features. The smaller number of features can better cover the feature space. ProTstab2 is not overfitted as evident from the performance on the blind test data, the scores are comparable to those in 10-fold CV (Table 4 and Table 5).

The PCC is 0.803 for ProTstab2 and the error measures are also good on the blind test data. The performance is clearly better than for the original ProTstab as well as another method that we could compare, SCooP. ProTstab2 is fast and reliable, it can be applied to proteins from any organism and run on large sets of proteins. We tested it on the proteomes of humans, mice, and zebrafish. A comparison of human–mouse orthologs indicated that sequence conservation did not correlate to stability differences.

The tool is freely available and allows the submission of sequence information in different formats.

## 4. Materials and Methods

### 4.1. Data Sets

The data were collected from two sources: ProTstab [14] and meltome atlas [17]. After deleting entries that contained ambiguous amino acids, we had 3500 records in four species from ProTstab, and 31,413 records from the meltome atlas for thirteen species. The final data set contained 34,913 records. Within this data set, we randomly divided the records into training and blind test sets. The data sets are available on the predictor website.

### 4.2. Features

In total, 6395 features were collected to describe protein properties. A total of 34,913 protein sequences were obtained from UniProtKB [26] and used to calculate the features.

The majority of the features were obtained with protr [27], an R package for generating various numerical representation schemes based on amino acid sequences. The package calculates eight descriptor groups composed of 22 types of commonly used descriptors. We obtained altogether 6295 features with protr.

A total of 140 features were extracted by RECON [28], which uses an algorithm based on Bader’s quantum theory of atoms in molecules. It provides molecular charge density-based electronic properties. RECON is available at http://reccr.chem.rpi.edu/Software/Protein-Recon/Protein-Recon-index.html (last accessed on 12 September 2022). This site does not work anymore, however, none of the features were selected for the final predictor.

Nineteen features were obtained with ProtDCal [29] which uses a hierarchical strategy. ProtParam [30] computes various physical and chemical parameters for protein sequences. The parameters include molecular weight, theoretical pI, amino acid composition, atomic composition, extinction coefficient, estimated half-life, instability index, aliphatic index, and grand average of hydropathy (GRAVY). A total of 28 features were obtained with ProtParam.

Amino acid group counts and frequencies. Amino acids were divided into 6 groups according to their physicochemical properties as follows: hydrophobic (V, I, L, F, M, W, Y, C), negatively charged (D, E), positively charged (R, K, H), conformational (G, P), polar (N, Q, S) and others (A, T) [31]. The numbers of amino acids in the 6 groups and their frequencies were calculated from the amino acid sequence, so there are 12 features in total.

Dipeptide counts. A total of 441 dipeptide counts were calculated in a window of 21 amino acids.

### 4.3. Algorithms

We started by choosing the best-performing algorithm among different methods.

DT is a non-parametric supervised learning method that has been used both for classification and regression. The method creates a model that predicts the value of a target variable based on simple decision rules inferred from the data features. Random forests (RFs) [32] is an extended bagging technique. The algorithm divides the training data into several partitions and builds a decision tree predictor for each partition. Finally, all the decision trees are merged into one predictor. RFs are resistant to overfitting.

SVR [33] is a widely used ML algorithm. SVR maps data into a high dimensional space and uses a kernel function. Training and testing of SVR with large data sets are very time-consuming. Therefore, we used linear support vector regression, which is similar to SVR with parameter kernel = “linear”, but is implemented according to liblinear [34] instead of libsvm [35]. This approach is more flexible in choosing penalties and loss functions and is therefore more suited to training predictors with large numbers of samples.

GBRT [36] can be used in many linear or non-linear prediction applications. The algorithm starts by building a decision tree model, then it gradually adds new decision trees to it to predict the residual value. Finally, the algorithm merges all the decision trees into one predictor. GBRT can process various types of data naturally, and it can deal robustly with outlier values because of its loss function. However, GBRT cannot be parallelized; therefore, it is slow to train.

XGBoost [37] is an optimized distributed gradient boosting library. XGBoost uses a parallel tree boosting (also known as gradient boosting decision tree (GBDT)) and gradient boosting machine (GBM).

LightGBM [38] is a gradient boosting framework that uses tree-based learning algorithms. It is a variant of GBDT model and utilizes gradient-based one-side sampling (GOSS) and exclusive feature bundling (EFB) to reduce the time complexity.

MLP regressor [39,40] optimizes the squared loss using the limited-memory Broyden, Fletcher, Goldfarb, and Shanno (LBFGS) algorithm or stochastic gradient descent (SGD). In our implementation, the hidden layer nodes were set to (20, 20, 20) and as the activation function was used Relu. Relu can solve exploding and vanishing gradient problems and keep the convergence rate in a steady state. LBFGS was used for the optimization.

All the algorithms were implemented in Python and run with default parameters. Scripts were written on Python 3.6. DT, RF, and SVR MLP were implemented in the standard scikit-learn (version 0.19.2). LGBM was implemented in the standard LightGBM (version 2.3.1 https://github.com/microsoft/LightGBM, accessed on 12 September 2022) and XGBoost was implemented in the standard xgboost (version 1.5.2 https://github.com/dmlc/xgboost, accessed on 12 September 2022).

### 4.4. Feature Selection

We tested two feature selection applications. Given an external estimator that assigns weights to features, the RFE [41] selects features recursively. Initially, the estimator is trained on the features and the importance of each feature is obtained and then the least important features are pruned. The procedure is recursively repeated until the desired number of features is reached.

RFECV performs RFE in a cross-validation loop to find the optimal number of features using RFE to evaluate the importance of the features. We used 3-fold cross-validation (CV) to select the optimal number of features.

### 4.5. Performance Assessment

The performances of the methods in regression were assessed with five measures. The PCC measures linear correlation between two sets of data. It is the covariance of two variables, divided by the product of their standard deviations, i.e., it is a normalized measurement of the covariance. PCC scores range from −1 to 1. It is calculated as

(1) $$\mathrm{PCC}=\frac{cov\left(X,Y\right)}{\sigma_{X}\sigma_{Y}}=\frac{\left(E\left[\left(X-\mu_{X}\right)\left(Y-\mu_{Y}\right)\right]\right)}{\sigma_{X}\sigma_{Y}},$$

where *cov* is the covariance, *σ_X_* is the standard deviation of *X*, *σ_Y_* is the standard deviation of *Y*, *µ_X_* is the mean of *X*, *µ_Y_* is the mean of *Y*, and *E* is the expectation.

The root mean square error (RMSE) is the measure of the differences between predicted and observed values:

(2) $$\mathrm{RMSE}=\sqrt{\frac{\sum_{i=1}^{N}{\left(y_{i}-x_{i}\right)}^{2}}{N}}.$$

The predicted value is *y_i_*, the experimental value is *x_i_*.

The mean absolute error (MAE) indicates the error between paired values for predictions and observations:

(3) $$\mathrm{MAE}=\frac{\sum_{i=1}^{N}\left|y_{i}-x_{i}\right|}{N},$$

where *y_i_* is the prediction and *x_i_* is the true value.

The mean squared error (MSE) measures the average of the squares of the errors, i.e., the average squared difference between the estimated values and the actual values

(4) $$\mathrm{MSE}=\frac{1}{N}\sum_{i=1}^{N}{\left(y_{i}-x_{i}\right)}^{2}.$$

A vector of *N* predictions is generated from a sample of *N* data points on all variables, and *x_i_* is the vector of observed values of the variable being predicted, with *y_i_* being the predicted values.

$R^{2}$ is the proportion of the variance in the dependent variable that is predictable from the independent variable(s). In regression, *R^2^* estimates how close the data are to the fitted regression line. The better the regression model, the closer the value is to 1.

(5) $$R^{2}=1-\frac{SS_{res}}{SS_{tot}}=1-\frac{\sum_{i}{\left(y_{i}-x_{i}\right)}^{2}}{\sum_{i}{\left(y_{i}-\bar{y}\right)}^{2}}$$

$SS_{tot}$ is the total sum of squares, $SS_{res}$ is the sum of squares of residuals. *y_i_* is the true value and *x_i_* is the prediction.

## Figures and Tables

**Table 1:** Numbers of proteins in species and data sets used for method development.

Species	OGT (°C)	ProTstab	Meltome Atlas	Total
*Oleispira antarctica*	15	0	1352	1352
*Caenorhabditis elegans*	20	0	3327	3327
*Arabidopsis thaliana*	25	0	2489	2489
*Danio rerio*	28	0	3362	3362
*Drosophila melanogaster*	28	0	1681	1681
*Bacillus subtilis*	30	0	1563	1563
*Saccharomyces cerevisiae*	30	706	1949	2655
*Homo sapiens*	37	984	5472	6456
*Escherichia coli*	37	729	1830	2559
*Mus musculus*	37	0	5800	5800
*Geobacillus stearothermophilus*	55	0	776	776
*Picrophilus torridus*	60	0	908	908
*Thermus thermophilus*	70	1081	904	1985
Total		3500	31,413	34,913

**Table 2:** Division of data sets for method training and testing.

	ProTstab	Meltome Atlas	ProTstab2
Blind test set	299	3144	3443
Training set	3201	28,269	31,470
Total	3500	31,413	34,913

**Table 3:** Performance of machine learning regression algorithms with all features in 10-fold cross-validation.

	DT	RF	SVR	GBRT	XGBoost	LightGBM	MLP
PCC	0.55	0.71	0.59	0.72	0.73	0.75	0.74
RMSE (°C)	10.21	7.58	8.88	7.43	7.42	7.11	7.26
*R* ^2^	0.09	0.50	0.31	0.52	0.52	0.56	0.54
MSE (°C)	104.22	57.46	78.88	55.15	55.07	50.50	52.87
MAE (°C)	7.45	5.60	6.68	5.51	5.49	5.27	5.35
Running time (s)	4076	23777	5258	27299	7977	673	7323

**Table 4:** Results for feature selection with RFE and RFECV in 10-fold cross-validation.

	RFE									RFECV
Number of features	50	100	200	300	500	1000	2000	3000	6935 (all)	1214
PCC	0.750	0.757	0.758	0.758	0.757	0.755	0.748	0.752	0.749	0.755
RMSE (°C)	7.083	7.021	6.991	6.992	7.001	7.027	7.114	7.062	7.104	7.032
*R* ^2^	0.563	0.570	0.574	0.574	0.573	0.570	0.559	0.565	0.560	0.569
MSE (°C)	50.189	49.304	48.887	48.906	49.028	49.396	50.614	49.878	50.485	49.469
MAE (°C)	5.282	5.228	5.197	5.196	5.204	5.217	5.277	5.240	5.271	5.221

**Table 5:** Comparison of the performances of ProTstab and ProTstab2 on blind test data set.

	ProTstab	ProTstab2
PCC	0.736	0.803
RMSE	9.636	9.097
MSE	93.581	82.752
MAE	8.158	6.934
*R^2^*	−0.850	0.580

**Table 6:** Comparison of the performances of ScooP and ProTstab2 for the 438 proteins in the blind test set that SCooP was able to predict.

	SCooP	ProTstab2
PCC	0.443	0.715
RMSE	16.926	7.605
MSE	286.480	57.837
MAE	13.867	5.682
*R* ^2^	−1.594	0.476

## Data Availability

The data sets and predictions used for the article are available at http://structure.bmc.lu.se/ProTstab2/ (accessed on 12 September 2022) and at http://8.133.174.28:8000/ProTstab2 (accessed on 12 September 2022).

## Supplementary Materials

The following supporting information can be downloaded at: https://www.mdpi.com/article/10.3390/ijms231810798/s1.
