# Supplementary material for: ProTstab2 for Prediction of Protein Thermal Stabilities
Source: Int J Mol Sci. 2022 Sep 16;23(18):10798. doi: 10.3390/ijms231810798 (PMC9505338; doi:10.3390/ijms231810798)
Supplement: Supplementary file 1 [file ijms-23-10798-s001.zip › ijms-1899396-supplementary.pdf]

ProTstab2 for Prediction of Protein Thermal Stabilities

Yang Yang [1,2], Jianjun Zhao [1], Lianjie Zeng [1] and Mauno Vihinen [3,*]

[1] School of Computer Science and Technology, Soochow University, Suzhou 215006, China

[2] Collaborative Innovation Center of Novel Software Technology and Industrialization, Nanjing 210000, China

[3] Department of Experimental Medical Science, BMC B13, Lund University, SE- 22184 Lund, Sweden

* Correspondence: mauno.vihinen@med.lu.se

Supplementary Materials

Supplementary Table S1. Types and numbers of protein descriptors calculated with protr.

Descriptor group	Descriptor	Number
Amino acid composition	Amino acid composition	20
	Dipeptide composition	400
	Normalized Moreau-Broto	240
Autocorrelation	Moran	240
	Geary	240
	Composition	21
CTD	Transition	21
	Distribution	105
Conjoint triad	Conjoint triad	343
Quasi-sequence-order	Sequence-order-coupling number	60
	Quasi-sequence-order descriptors	100
Pseudo-amino acid composition	Type I	50
	Type II	80
Proteochemometric descriptors	Principal component analysis (based on 2D and 3D molecular descriptors)	4025
	Factor analysis (amino acid properties based)	175
	Multidimensional scaling (amino acid properties based)	175

Supplementary Table S2. The 200 informative features used to train ProTstab2, their origin and importance.

Rank	Feature name	Description	Origin	Importance
1	fre(G6_5)	Group5_frequency	Manual	88
2	I	Amino acid composition: I	protr	67
3	Schneider.Xr.Q	Quasi-sequence-order (QSO) descriptor	protr	40
4	Grantham.Xr.I	Quasi-sequence-order (QSO) descriptor	protr	40
5	solventaccess.Group1	Composition descriptor of CTD descriptors	protr	38
6	Schneider.Xr.C	Quasi-sequence-order (QSO) descriptor	protr	35
7	Grantham.Xr.Y	Quasi-sequence-order (QSO) descriptor	protr	31
8	Xc1.C	Pseudo amino acid composition (PseAAC) descriptor	protr	31
9	AAACF_scl5.4.lag1	Scales-based descriptors with AAACF of molecular descriptors	protr	28
10	Theoretical pI	Theoretical pI	Expasy	27
11	Schneider.Xr.K	Quasi-sequence-order (QSO) descriptor	protr	26
12	Y	Amino acid composition: Y	protr	25
13	ER	Dipeptide composition frequency: ER	protr	25
14	prop7.G3.residue25	Composition descriptor of the CTD descriptors	protr	25
15	Schneider.Xr.D	Quasi-sequence-order (QSO) descriptor	protr	24
16	Schneider.Xr.E	Quasi-sequence-order (QSO) descriptor	protr	24
17	prop5.G1.residue0	Composition descriptor of the CTD descriptors	protr	23
18	VS246	Conjoint triad	protr	23
19	Schneider.Xr.Y	Quasi-sequence-order (QSO) descriptor	protr	23
20	Grantham.Xr.C	Quasi-sequence-order (QSO) descriptor	protr	23
21	A	Amino acid composition: A	protr	22
22	prop7.Tr1331	Composition descriptor of the CTD descriptors	protr	22
23	Grantham.Xr.Q	Quasi-sequence-order (QSO) descriptor	protr	22
24	AAEigIdx_ scl4.2.lag1	Scales-based descriptors with AAEigIdx of molecular descriptors	protr	22
25	C	Amino acid composition: C	protr	21
26	Q	Amino acid composition: Q	protr	21
27	Schneider.Xr.I	Quasi-sequence-order (QSO) descriptor	protr	20
28	Grantham.Xr.S	Quasi-sequence-order (QSO) descriptor	protr	20

29	Grantham.Xr.V	Quasi-sequence-order (QSO) descriptor	protr	20
30	AAGeom_scl4.1.lag1	Scales-based descriptors with AAGeom of molecular descriptors	protr	20
31	AARandic_scl1.4.lag1	Scales-based descriptors with AARandic of molecular descriptors	protr	20
32	D	Amino acid composition: D	protr	19
33	EV	Dipeptide composition frequency: EV	protr	19
34	CIDH920105.lag6	Normalized Moreau-Broto autocorrelation	protr	19
35	polarizability.Group2	Composition descriptor of the CTD descriptors	protr	19
36	Schneider.Xr.S	Quasi-sequence-order (QSO) descriptor	protr	19
37	Grantham.Xr.R	Quasi-sequence-order (QSO) descriptor	protr	19
38	Pc1.R	Amphiphilic Pseudo-Amino acid composition	protr	19
39	AAMOE2D_scl5.1.lag1	Scales-based descriptors with AAMOE2D of molecular descriptors	protr	19
40	AAConst_ scl4.5.lag4	Scales-based descriptors with AAConst of molecular descriptors	protr	19
41	AAWHIM_scl5.2.lag1	Scales-based descriptors with AAWHIM of molecular descriptors	protr	19
42	R	Amino acid composition: R	protr	18
43	QE	Dipeptide composition frequency: QE	protr	18
44	prop3.G2.residue0	Composition descriptor of the CTD descriptors	protr	18
45	AAEigIdx_ scl4.lag3	Scales-based descriptors with AAEigIdx of molecular descriptors	protr	18
46	AAFGC_scl3.4.lag3	Scales-based descriptors with AAFGC of molecular descriptors	protr	18
47	AAInfo_ scl3.5.lag1	Scales-based descriptors with AAInfo of molecular descriptors	protr	18
48	ID	Dipeptide composition frequency: ID	protr	17
49	IE	Dipeptide composition frequency: IE	protr	17
50	IK	Dipeptide composition frequency: IK	protr	17
51	secondarystruct.Group3	Composition descriptor of the CTD descriptors	protr	17
52	Xc1.K	Pseudo-amino acid composition	protr	17
53	Pc1.A	Amphiphilic pseudo-amino acid composition	protr	17

54	AAMOE2D_scl5.2.lag4	Scales-based descriptors with AAMOE2D of molecular descriptors	protr	17
55	AAGeom_scl4.3.lag4	Scales-based descriptors with AAGeom of molecular descriptors	protr	17
56	AAInfo_scl1.5.lag2	Scales-based descriptors with AAInfo of molecular descriptors	protr	17
57	AARandic_scl3.2.lag4	Scales-based descriptors with AARandic of molecular descriptors	protr	17
58	fre(G6_4)	6 amino acid group frequencies	Manual	16
59	N	Amino acid composition: N	protr	16
60	FE	Dipeptide composition frequency: FE	protr	16
61	CIDH920105.lag5	Moran autocorrelation	protr	16
62	CHAM820102.lag1	Moran autocorrelation	protr	16
63	Grantham.Xr.N	Quasi-sequence-order (QSO) descriptor	protr	16
64	Pc1.Y	Amphiphilic pseudo-amino acid composition	protr	16
65	AAMOE2D_ scl1.4.lag2	Scales-based descriptors with AAMOE2D of molecular descriptors	protr	16
66	AADescAll_scl5.4.lag1	Scales-based descriptors with AADescAll of molecular descriptors	protr	16
67	AA3DMoRSE_scl4.lag1	Scales-based descriptors with AA3DMoRSE of molecular descriptors	protr	16
68	AAConst_scl1.3.lag3	Scales-based descriptors with AAConst of molecular descriptors	protr	16
69	AAConst_ scl4.5.lag3	Scales-based descriptors with AAConst of molecular descriptors	protr	16
70	AAFGC_scl5.4.lag1	Scales-based descriptors with AAFGC of molecular descriptors	protr	16
71	AAInfo_scl3.lag5	Scales-based descriptors with AAInfo of molecular descriptors	protr	16
72	S	Amino acid composition: S	protr	15
73	EA	Dipeptide composition frequency: EA	protr	15
74	LA	Dipeptide composition frequency: LA	protr	15
75	II	Dipeptide composition frequency: II	protr	15
76	CHAM820102.lag23	Moran autocorrelation	protr	15

77	CHAM820101.lag26	Geary autocorrelation	protr	15
78	VS164	Conjoint triad	protr	15
79	Grantham.Xr.A	Quasi-sequence-order (QSO) descriptor	protr	15
80	Grantham.Xr.F	Quasi-sequence-order (QSO) descriptor	protr	15
81	AA2DACOR_scl4.1.lag1	Scales-based descriptors with AA2DACOR of molecular descriptors	protr	15
82	AABurden_scl5.4.lag1	Scales-based descriptors with AABurden of molecular descriptors	protr	15
83	AAMolProp_ scl1.lag1	Scales-based descriptors with AAMolProp of molecular descriptors	protr	15
84	AARDF_scl1.4.lag1	Scales-based descriptors with AARDF of molecular descriptors	protr	15
85	prop3.G1.residue25	Composition descriptor of the CTD descriptors	protr	14
86	AAMOE3D_ scl5.2.lag6	Scales-based descriptors with AAMOE3DF of molecular descriptors	protr	14
87	AAEigIdx_scl4.lag7	Scales-based descriptors with AAEigIdx of molecular descriptors	protr	14
88	AAEigIdx_scl2.4.lag4	Scales-based descriptors with AAEigIdx of molecular descriptors	protr	14
89	AATopoChg_scl5.1.lag1	Scales-based descriptors with AATopoChg of molecular descriptors	protr	14
90	AAWalk_scl2.5.lag4	Scales-based descriptors with AAWalk of molecular descriptors	protr	14
91	H	Amino acid composition: H	protr	13
92	EE	Dipeptide composition frequency: EE	protr	13
93	CIDH920105.lag11	Normalized Moreau-Broto autocorrelation	protr	13
94	Pc1.N	Amphiphilic pseudo-amino acid composition	protr	13
95	AA3DMoRSE_scl2.5.lag1	Scales-based descriptors with AA3DMoRSE of molecular descriptors	protr	13
96	AAGeom_scl3.2.lag1	Scales-based descriptors with AAGeom of molecular descriptors	protr	13
97	AAInfo_scl2.lag2	Scales-based descriptors with AAInfo of molecular descriptors	protr	13
98	AAInfo_scl2.lag3	Scales-based descriptors with AAInfo of molecular descriptors	protr	13

99	AARandic_scl4.lag1	Scales-based descriptors with AARandic of molecular descriptors	protr	13
100	AATopoChg_scl5.1.lag3	Scales-based descriptors with AATopoChg of molecular descriptors	protr	13
101	AAWHIM_scl4.5.lag1	Scales-based descriptors with AAWHIM of molecular descriptors	protr	13
102	fre(G6_1)	6 amino acid group frequencies	Manual	12
103	PE	Dipeptide composition frequency: PE	protr	12
104	PG	Dipeptide composition frequency: PG	protr	12
105	KF	Dipeptide composition frequency: KF	protr	12
106	PV	Dipeptide composition frequency: PV	protr	12
107	CIDH920105.lag8	Normalized Moreau-Broto autocorrelation	protr	12
108	prop3.Tr1221	Composition descriptor of the CTD descriptors	protr	12
109	prop5.G3.residue0	Composition descriptor of the CTD descriptors	protr	12
110	prop6.G1.residue25	Composition descriptor of the CTD descriptors	protr	12
111	VS126	Conjoint triad	protr	12
112	VS136	Conjoint triad	protr	12
113	Xc2.lambda.18	Pseudo-amino acid composition	protr	12
114	Pc1.F	Amphiphilic pseudo-amino acid composition	protr	12
115	AACPSA_scl4.lag3	Scales-based descriptors with AACPSA of molecular descriptors	protr	12
116	AAConn_scl2.lag5	Scales-based descriptors with AAConn of molecular descriptors	protr	12
117	AAEigIdx_ scl4.lag5	Scales-based descriptors with AAEigIdx of molecular descriptors	protr	12
118	AAFGC_scl1.5.lag1	Scales-based descriptors with AAFGC of molecular descriptors	protr	12
119	AARDF_scl4.lag1	Scales-based descriptors with AARDF of molecular descriptors	protr	12
120	AATopo_scl3.4.lag1	Scales-based descriptors with AATopo of molecular descriptors	protr	12
121	AATopo_scl5.1.lag4	Scales-based descriptors with AATopo of molecular descriptors	protr	12

122	AATopoChg_scl4.1.lag5	Scales-based descriptors with AATopoChg of molecular descriptors	protr	12
123	AAWalk_scl2.4.lag6	Scales-based descriptors with AAWalk of molecular descriptors	protr	12
124	YG	Dipeptide composition frequency: YG	protr	11
125	LI	Dipeptide composition frequency: LI	protr	11
126	DP	Dipeptide composition frequency: DP	protr	11
127	BHAR880101.lag7	Normalized Moreau-Broto autocorrelation	protr	11
128	CHAM810101.lag18	Normalized Moreau-Broto autocorrelation	protr	11
129	VS237	Conjoint triad	protr	11
130	Schneider.Xr.L	Quasi-sequence-order (QSO) descriptor	protr	11
131	Xc2.lambda.2	Pseudo-amino acid composition	protr	11
132	AA3DMoRSE_scl3.lag4	Scales-based descriptors with AA3DMoRSE of molecular descriptors	protr	11
133	AAEdgeAdj_scl4.1.lag1	Scales-based descriptors with AAEdgeAdj of molecular descriptors	protr	11
134	AAEigIdx_scl5.4.lag1	Scales-based descriptors with AAEigIdx of molecular descriptors	protr	11
135	AAGeom_scl3.4.lag2	Scales-based descriptors with AAGeom of molecular descriptors	protr	11
136	AAGeom_scl2.3.lag5	Scales-based descriptors with AAGeom of molecular descriptors	protr	11
137	AAGeom_scl5.1.lag6	Scales-based descriptors with AAGeom of molecular descriptors	protr	11
138	AAGETAWAY_scl2.lag3	Scales-based descriptors with AAGETAWAY of molecular descriptors	protr	11
139	AAInfo_scl5.3.lag1	Scales-based descriptors with AAInfo of molecular descriptors	protr	11
140	AARandic_scl5.lag6	Scales-based descriptors with AARandic of molecular descriptors	protr	11
141	AARDF_scl3.4.lag6	Scales-based descriptors with AARDF of molecular descriptors	protr	11
142	AARDF_scl1.3.lag7	Scales-based descriptors with AARDF of molecular descriptors	protr	11

143	AAWalk_scl2.5.lag3	Scales-based descriptors with AAWalk of molecular descriptors	protr	11
144	AAWHIM_scl3.lag2	Scales-based descriptors with AAWHIM of molecular descriptors	protr	11
145	AAWHIM_scl2.4.lag1	Scales-based descriptors with AAWHIM of molecular descriptors	protr	11
146	count(E to A)	Dipeptide composition count: EA	Manual	10
147	P	Amino acid composition: P	protr	10
148	SA	Dipeptide composition frequency: SA	protr	10
149	NI	Dipeptide composition frequency: NI	protr	10
150	SI	Dipeptide composition frequency: SI	protr	10
151	AV	Dipeptide composition frequency: AV	protr	10
152	CHAM820102.lag25	Geary autocorrelation	protr	10
153	secondarystruct.Group2	Composition descriptor of the CTD descriptors	protr	10
154	prop1.Tr2332	Composition descriptor of the CTD descriptors	protr	10
155	prop6.Tr1331	Composition descriptor of the CTD descriptors	protr	10
156	VS322	Conjoint triad	protr	10
157	VS215	Conjoint triad	protr	10
158	Schneider.Xr.R	Quasi-sequence-order (QSO) descriptor	protr	10
159	Schneider.Xr.M	Quasi-sequence-order (QSO) descriptor	protr	10
160	Xc2.lambda.5	Pseudo-amino acid composition	protr	10
161	AACPSA_scl2.lag5	Scales-based descriptors with AACPSA of molecular descriptors	protr	10
162	AACPSA_scl5.3.lag4	Scales-based descriptors with AACPSA of molecular descriptors	protr	10
163	AA2DACOR_scl1.5.lag1	Scales-based descriptors with AA2DACOR of molecular descriptors	protr	10
164	AAACF_scl5.1.lag3	Scales-based descriptors with AAACF of molecular descriptors	protr	10
165	AAFGC_scl3.5.lag1	Scales-based descriptors with AAFGC of molecular descriptors	protr	10
166	AAGETAWAY_scl4.1.lag3	Scales-based descriptors with AAGETAWAY of molecular descriptors	protr	10

167	AAInfo_scl2.lag7	Scales-based descriptors with AAInfo of molecular descriptors	protr	10
168	AAMolProp_scl4.3.lag7	Scales-based descriptors with AAMolProp of molecular descriptors	protr	10
169	AAWalk_scl5.lag1	Scales-based descriptors with AAWalk of molecular descriptors	protr	10
170	AAWHIM_scl4.lag1	Scales-based descriptors with AAWHIM of molecular descriptors	protr	10
171	AAWHIM_scl4.1.lag5	Scales-based descriptors with AAWHIM of molecular descriptors	protr	10
172	DQ	Dipeptide composition frequency: DQ	protr	9
173	YI	Dipeptide composition frequency: YI	protr	9
174	CIDH920105.lag4	Normalized Moreau-Broto autocorrelation	protr	9
175	BHAR880101.lag19	Geary autocorrelation	protr	9
176	VS444	Conjoint triad	protr	9
177	AAConst_scl4.5.lag6	Scales-based descriptors with AAConst of molecular descriptors	protr	9
178	AARDF_scl5.2.lag1	Scales-based descriptors with AARDF of molecular descriptors	protr	9
179	scl4.3.lag1	Scales-based descriptors derived by multidimensional scaling	protr	9
180	M	Amino acid composition: M	protr	8
181	QG	Dipeptide composition frequency: QG	protr	8
182	AI	Dipeptide composition frequency: AI	protr	8
183	GP	Dipeptide composition frequency: GP	protr	8
184	KS	Dipeptide composition frequency: KS	protr	8
185	CIDH920105.lag21	Normalized Moreau-Broto autocorrelation	protr	8
186	BHAR880101.lag12	Normalized Moreau-Broto autocorrelation	protr	8
187	BHAR880101.lag30	Normalized Moreau-Broto autocorrelation	protr	8
188	CHAM820101.lag13	Moran autocorrelation	protr	8
189	AACPSA_scl4.lag1	Scales-based descriptors with AACPSA of molecular descriptors	protr	8
190	AAEdgeAdj_scl5.1.lag1	Scales-based descriptors with AAEdgeAdj of molecular descriptors	protr	8

191	VC	Dipeptide composition frequency: VC	protr	7
192	VS426	Conjoint triad	protr	7
193	AAEigId_scl5.2.lag6	Scales-based descriptors with AAEigId of molecular descriptors	protr	7
194	AAInfo_scl3.lag4	Scales-based descriptors with AAInfo of molecular descriptors	protr	7
195	AAInfo_scl2.1.lag4	Scales-based descriptors with AAInfo of molecular descriptors	protr	7
196	ML	Dipeptide composition frequency: ML	protr	6
197	AAACF_scl3.lag1	Scales-based descriptors with AAACF of molecular descriptors	protr	6
198	AAMolProp_scl1.lag2	Scales-based descriptors with AAMolProp of molecular descriptors	protr	6
199	AAWalk_scl2.4.lag7	Scales-based descriptors with AAWalk of molecular descriptors	protr	6
200	secondarystruct.Group1	Composition descriptor of the CTD descriptors	protr	5
